# The impact of life satisfaction on acculturation and institutional recommendation among international students in China: does acculturative stress matter?

**DOI:** 10.3389/fpsyg.2025.1584210

**Published:** 2025-07-02

**Authors:** Zhongli Wu, Hazem Ali, Chen Yupeng

**Affiliations:** ^1^School of International Studies, Yiwu Industrial and Commercial College, Jinhua, China; ^2^School of Economics and Management, Yiwu Industrial and Commercial College, Jinhua, China; ^3^School of International Education, Yiwu Industrial and Commercial College, Jinhua, China

**Keywords:** international students’ experiences, life satisfaction, acculturation, institutional recommendation, acculturative stress

## Abstract

**Introduction:**

Given the substantial contributions of international students to cultural diversity and institutional development, examining the antecedents of their life satisfaction, acculturation status, and willingness to recommend their institutions has garnered significant attention from policymakers and scholars. This study had two main objectives: 1) to assess the association between international students’ experiences and their life satisfaction, and 2) to examine the impact of life satisfaction on acculturation and institutional recommendation, with a particular focus on the moderating role of acculturative stress.

**Methods:**

A total of 281 international students from 13 universities in China participated in the study by completing self-administered questionnaires.

**Results:**

Regression results showed that international students’ socio-cultural, accommodation, health and safety, and support service experiences were positively related to life satisfaction. In contrast, academic experiences were negatively related to life satisfaction, while discrimination experiences showed no significant impact. In addition, life satisfaction was positively associated with acculturation and institutional recommendation. Further, the moderating results showed that acculturative stress weakens the positive relationship between life satisfaction and acculturation.

**Discussion:**

Our findings underscore the importance of improving academic environments, enhancing students’ non-academic experiences, and addressing acculturative stress to enhance the acculturation state and positive institutional recommendations of international students.

## 1 Introduction

The Chinese government has implemented several proactive policies aimed at enhancing the quality of services for international students and sustaining growth in the international education market. These measures include increasing the availability of scholarships, expanding English-language programs (Ding, 2016; Wen et al., 2018), and establishing educational cooperation and exchange agreements with 188 countries and regions (Zhu et al., 2022). Despite China’s ranking as Asia’s top study-abroad destination, the objectives of the internationalization of higher education in China are not well achieved (Mulvey, 2020). However, the increasing number of international students in China does not necessarily indicate high life satisfaction or a greater likelihood of recommending their institutions, as many face emotional challenges such as depression and loneliness (Li et al., 2021). Hence, several scholars called for more research to investigate international students’ reactions to their experiences in the host country (Wekullo, 2019), especially in non-native English countries (Calikoglu, 2018).

Life satisfaction constitutes a fundamental aspect of international students’ psychological well-being during acculturation (Karaman and Watson, 2017; Jiang et al., 2020; Peng et al., 2023). Given their global and economic importance, numerous researchers advocated that examining the experience of international students should be regarded as an essential antecedent of their life satisfaction (Wekullo, 2019). Extant research indicated that the level of international students’ life satisfaction in Western countries like the US, the UK, and Australia is higher than in China (Ding, 2016; Wen and Hu, 2019) while a limited number of studies paid attention to understanding the international students’ life satisfaction in China (Zhang and Zeng, 2023).

While studying abroad offers clear benefits such as language acquisition and social capital development, the acculturation process often brings psychological and adjustment challenges for international students (Smith and Khawaja, 2011). Various studies examined the antecedents of international students’ acculturation process (Song and Xia, 2021; Zhang and Zeng, 2023), and focused mainly on the Western context, while limited studies focused on investigating the acculturation state of international students in the Chinese context (Gao and Hua, 2021; Zhang and Zeng, 2023). Extant acculturation research underlined that international students who encounter cultural shocks experience psychological stress and obstacles during their acculturation process due to differences in culture, language, and education system (Gao and Hua, 2021; Li et al., 2021; Zhang and Zeng, 2023). Several scholars called for more research on understanding the acculturation of international students in China (Luo et al., 2023; Grigoryev and Berry, 2017; Lai et al., 2023).

Amid growing global competition for international student enrollment, it is essential to critically examine the factors shaping their institutional evaluation and selection decisions (Ammigan et al., 2020). Foreign students with high life satisfaction in the host country are more likely to recommend their universities and share positive word-of-mouth (Mavondo et al., 2004; Padlee and Reimers, 2015). While a multicultural study environment may positively influence international students’ overall satisfaction, it may hurt the likelihood of recommendation (Ammigan et al., 2020).

Recent studies have increasingly examined acculturative stress among international students, focusing on its effects on academic performance, quality of life, and psychological well-being (Benita and Supriya, 2016; Kristiana et al., 2022). According to Gyamerah et al. (2024), international students often lack sufficient support from faculty and administrators, and that self-efficacy alone may not be enough to manage acculturative stress.

This research advances the literature on international students’ behavioral outcomes—satisfaction, acculturation, and institutional recommendation—in three key ways. First, drawing on Social Exchange Theory and Berry’s acculturation model, it proposes a comprehensive framework linking students’ perceived experiences to life satisfaction and, in turn, to their acculturation and institutional recommendation, while accounting for the moderating role of acculturative stress. Second, existing empirical research on international students’ life satisfaction in China has largely concentrated on major cities such as Shanghai and Beijing (Ding, 2016; Wen et al., 2018). Yet, students in less developed cities may encounter different academic and sociocultural challenges that impact their satisfaction. Additionally, the literature on life satisfaction and acculturation remains dominated by qualitative and theoretical reviews, with limited empirical studies available (Anderson and Guan, 2018; Peng et al., 2023). Third, within the Chinese context, prior research has largely focused on the internationalization of higher education and enrollment growth, with limited attention to international students’ experiences, life satisfaction, and acculturation (Jiang et al., 2020).

This study underscores the importance of distinguishing between evaluation (life satisfaction) and behavioral intention (acculturation and institutional recommendation). Understanding this distinction is critical, as student satisfaction with their educational experience does not always translate into recommending the institution. While satisfaction may stem from various factors such as teaching quality and campus facilities recommendations are more strongly influenced by perceived value and future employability (Ghorbanzade et al., 2019).

## 2 Theory and hypotheses

This study is grounded in Social Exchange Theory (SET) by Blau (1964) and Berry (2003) acculturation theory to examine the direct impact of international students’ experiences on their life satisfaction; and the influence of life satisfaction on their acculturation state and their institutional recommendations while considering the indirect influence of acculturative stress. SET provides a framework for understanding how international students evaluate their experiences based on perceived costs and benefits. According to the SET, individuals are more likely to engage in reciprocal behavior such as high acculturation and positive institutional recommendations when they sense benefits from their surroundings. Although studying abroad offers significant benefits like enhanced self-growth and confidence (Taušová et al., 2019), international students often face challenges such as discrimination, unfamiliar academic expectations, language barriers, limited social support, homesickness, loneliness, and weak identification with the host culture (Chiang, 2015; Taušová et al., 2019; Bai et al., 2023). Hence, this study argues that satisfied students reciprocate the positive experience by recommending their institutions in the host country to future students.

Berry’s Acculturation Theory offers a cultural and psychological framework for understanding how international students adapt to new cultural environments and manage stress through various coping strategies. It distinguishes between acculturation: the cultural and psychological changes resulting from intercultural contact (Berry, 2003) and adaptation: which refers to the outcome of acculturation, reflecting the degree of fit between the individual and the host culture (Berry, 1997).

Researchers typically categorize acculturation into two models. The first is the unidirectional model, which conceptualizes acculturation as a linear process progressing along a continuum from the maintenance of one’s heritage culture on one end to full immersion in the host culture on the other (Umami et al., 2022). This model was criticized for preventing migrants from acculturating to both the host and origin cultures concurrently (Umami et al., 2022). The second model is known as bidimensional acculturation which argues that the maintenance of the original national culture and the acceptance of host culture must be viewed as two distinct dimensions (Berry, 2006). This study builds on the second model and argues that the decrease or growth of international students in the host or home country does not affect the other.

The former acculturation research proposed two dimensions of acculturation orientations to explain how immigrants and sojourners interact with their heritage and international cultures: (a) the desire to preserve the heritage culture and (b) the desire to interact with others in the dominant culture (Berry, 1997, 2005). This study defines international students’ acculturation as the set of tactics and strategies employed to navigate and adapt to the host country’s cultural environment while retaining aspects of their original culture. The current study examined the relationship between international students’ life satisfaction and their acculturation in China. Although students may appreciate the resources, engagement opportunities, and institutional efforts to foster diversity and multiculturalism on campus, adapting to new academic, social, and cultural settings can still be challenging and stressful (Mesidor and Sly, 2016). These cultural differences can create difficulties, and when reflecting on their experiences, students may be less willing to recommend such environments to others.

### 2.1 International students’ experiences and life satisfaction

**Figure 1:**
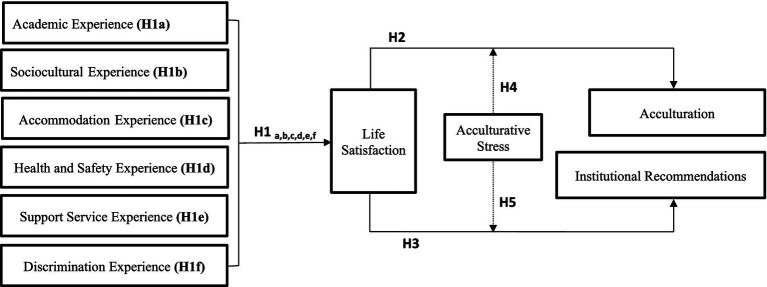
Research model.

Researchers presented several factors affecting international student’s satisfaction such as satisfaction with the program, lecturers’ instruction, the institution, campus facilities, student support offered, one’s learning, the overall university experience, and university life in general (Wong and Chapman, 2023). Research on understanding the relationship between experiences and life satisfaction of international students in China focused mainly on the academic and socio-cultural dimensions (Qadeer et al., 2021). The current study contends that educational and non-educational experiences differ among international students from various regions. It contributes to the existing literature on international student satisfaction by investigating the relationship between perceived experiences—specifically academic, accommodation, socio-cultural, health and safety, discrimination, and support services—and students’ overall life satisfaction. The proposed research framework is illustrated in Figure 1.

#### 2.1.1 Academic experience and international student life satisfaction

Numerous scholars highlighted the academic challenges faced by international students, including adapting to the host country’s academic system, selecting appropriate courses, communicating with lecturers and supervisors, understanding lectures and assessment methods (Hussain and Shen, 2019), as well as coping with strict examinations, uniform teaching styles, and rigid schedules (Nikula and Sibley, 2020). International students who struggle to understand and adapt to a new academic system and fail to develop necessary skills often face significant challenges in achieving their academic goals (Hussain and Shen, 2019). Research has shown that international students’ overall university experience is strongly linked to their level of academic satisfaction (Ammigan and Jones, 2018), with faculty accessibility and teaching quality identified as key drivers of satisfaction (Arambewela and Hall, 2009). This study argues that academic experiences, such as teaching quality, curriculum relevance, access to resources, and faculty interaction, play a crucial role in shaping international students’ overall life satisfaction. Accordingly, the first sub-hypothesis is proposed as:

*H1a*: International students' life satisfaction is positively linked to academic experiences.

#### 2.1.2 Sociocultural experience and international student life satisfaction

The sociocultural experience involves the perceptions of international students regarding their treatment during their studies abroad, as well as the cultural challenges encountered during their studies (Wen et al., 2018). Research on international students in China has shown that many face challenges in adapting to local socio-cultural dynamics (Wen et al., 2018). Given China’s distinct sociocultural context compared to Western countries, international students may encounter unique acculturation challenges (Zhang and Zeng, 2023). In contrast, studies in Western contexts like the U. S., Australia, and the U. K. have found that building social networks positively influences international students’ satisfaction (Ammigan, 2019). This research predicts that international students’ positive sociocultural experiences, such as participating in cultural exchanges, building friendships, and engaging in campus activities, foster a sense of belongingness and enhance their life satisfaction. Therefore, the second sub-hypothesis is stated as follows:

*H1b*: international students' life satisfaction is positively linked to their sociocultural experiences.

#### 2.1.3 Accommodation experience and international student life satisfaction

The quality of accommodations plays a significant role in improving international students’ experience in a host country (Ammigan and Jones, 2018). Most International students in China live at separate university hostels or other accommodations and coursework which may hinder their interaction with local students (Ding, 2016; Tian and Lowe, 2018; Wen et al., 2018). In the Chinese context, researchers advocated the importance of accommodations in shaping the satisfaction of international students (Tian and Lowe, 2018; Wen and Hu, 2019). In this regard, some concerns were identified by international students such as slow Internet, lack of single rooms, restricted visitor time, outdated dorm facilities, the separation of foreign students’ accommodations, and unprofessional behavior from dorm staff (Tian and Lowe, 2018; Wen and Hu, 2019). This study hypothesizes that accommodation quality, specifically comfortable and affordable housing, positively contributes to international students’ life satisfaction by ensuring a stable and supportive living environment. Accordingly, the third sub-hypothesis is proposed as follows:

*H1c*: international students' life satisfaction is positively related to their accommodation experiences.

#### 2.1.4 Health and safety experience and international student life satisfaction

The issue of international students’ safety become a major concern in the field of international education, especially after the deadly attacks on international students in several countries such as the USA, Australia, and India (Chelliah et al., 2019). Hence, international students and their family members may worry about potential safety and health issues in the host country (Arambewela and Hall, 2009). According to Zhang and Goodson (2011), there is a strong correlation between international students’ evaluations of life satisfaction and their acculturation experiences in the host nation. For instance, individuals can face unforeseen obstacles and difficulties adjusting to an international culture, which could lead to mental or psychological strain. Considering these factors and ensuring the safety of international students play a fundamental role in influencing their life satisfaction (Chelliah et al., 2019). The current study predicts that access to reliable healthcare, a safe campus, and clear safety protocols can enhance students’ sense of security, contributing positively to their satisfaction. The fourth sub-hypothesis is set as follows:

*H1d*: international students' life satisfaction is positively associated with their health and safety experiences.

#### 2.1.5 Support service experience and international student life satisfaction

Chinese universities typically maintain international students’ offices to assist foreign students with both academic and non-academic issues. Several studies have reported low levels of life satisfaction among international students in China (Ding, 2016; Tian and Lowe, 2018; Wen and Hu, 2019). Key concerns include inefficient administrative services—particularly regarding class schedules, final exams, and academic activities—as well as university websites that fail to meet international students’ information needs due to technical issues and complex navigation. Additionally, limited support with practical matters, such as opening bank accounts, airport pickups, and housing assistance, has been highlighted as a significant drawback (Ding, 2016).

This research defines support services experience as the international students’ evaluation of their interactions with the international student office, encompassing staff responsiveness, friendliness, and helpfulness in addressing student needs. Several studies have demonstrated that the availability and quality of campus support services significantly affect international students’ life satisfaction (Alemu and Cordier, 2017; Ammigan and Jones, 2018). Another study showed a favorable correlation between university assistance, a decrease in psychological stress, and an improvement in life satisfaction (Cho and Yu, 2015). The current research predicts that support services, including academic advising and counseling, can foster international students’ well-being and belongingness which eventually increases their life satisfaction. Accordingly, the fifth sub-hypothesis is stated as follows:

*H1e*: international students' life satisfaction is positively linked to support service experiences.

#### 2.1.6 Discrimination experience and international student life satisfaction

Research showed that discrimination negatively influences international students’ satisfaction, especially in building friendships in the host country (Van Horne et al., 2018; Wekullo, 2019). For instance, administrators’ attitudes toward international students appeared to be influenced by the perceived wealth of their countries of origin; and black students were more susceptible to racist attitudes, though this was not pervasive (Tian and Lowe, 2018). In addition, international students with religious commitments may struggle harder to maintain religious activities and make relationships, which could lead to a significant amount of stress (Yu et al., 2014). For instance, Muslim international students claimed that they could only pray in the hallways of their dorms and not in public (Tian and Lowe, 2018). This study argues that international students who experience discriminatory behavior, whether based on race, nationality, or language, are likely to have reduced life satisfaction as discrimination can create feelings of exclusion, alienation, and distress. Accordingly, the sixth sub-hypothesis is stated as follows:

*H1f*: international students' life satisfaction is negatively related to discrimination experiences.

### 2.2 Life satisfaction and acculturation

Numerous scholars indicate that the psychological acculturation of international students is associated with their higher-level life satisfaction (Peng et al., 2023). Student life satisfaction has a positive impact on enhancing international students’ acculturation in China by providing a foundation of emotional and psychological well-being that supports their acculturation (Qadeer et al., 2021). During their acculturation, international students face some challenges such as inadequate student-faculty interactions on campus (Wen et al., 2018), cultural differences in the relationship between lecturers’ behaviors and international students, lack of academic staff with adequate English skills, and misunderstandings of teaching methodologies (Jiang et al., 2021). Hence, researchers suggested different ways to enhance acculturation status such as creating campus activities to incorporate international students in and enhance international students’ awareness of the challenges faced in a new environment (Lee and Rice, 2007), responding to international students’ linguistic and cultural background (Siczek, 2015). A student’s motivation to investigate and adjust to new cultural standards is increased when they are satisfied, which improves acculturation (Jiang et al., 2020). Guided by relevant prior research, the second hypothesis is set as follows:

*H2*: higher life satisfaction positively influences the acculturation of international students.

### 2.3 Student life satisfaction and institutional recommendation

Life satisfaction among international students and their likelihood of recommending an institution may be influenced by the opinions and suggestions of friends, family members, and acquaintances when selecting a study destination and institution (Mavondo et al., 2004). Several researchers argued that students’ life satisfaction has a significant influence on spreading positive word-of-mouth and enhancing the institutions’ reputation (Clemes et al., 2008; Wilkins and StephensBalakrishnan, 2013; Kairat et al., 2024).

Students’ institutional recommendations are positively influenced by their satisfaction with institutional support and alignment with the institution’s values (Kairat et al., 2024). International students’ overall satisfaction is largely driven by teaching-related factors, such as program organization and lecture quality, while study-related factors like English language support and employability skills are more strongly linked to their institutional recommendations (Ammigan et al., 2020). Dissatisfaction, on the other hand, can lead to disengagement or less interaction with the host culture, impeding acculturation (Jiang et al., 2020). This research argues that the life satisfaction of international students can be translated into a strong willingness to recommend the institution to others. Hence, the third hypothesis is stated as follows:

*H3*: higher life satisfaction positively influences the institutional recommendation of international students.

### 2.4 The moderating role of acculturative stress

Researchers presented different predictors of acculturative stress such as the risk of experiencing financial, transportation, and accommodation issues (Smith and Khawaja, 2011). Numerous studies have demonstrated that acculturative stress is positively associated with psychosomatic symptoms, poor adjustment, and psychological distress, including depression, anxiety, hopelessness, and a sense of alienation (Driscoll and Torres, 2022). Some individuals may experience negative emotions and reduced motivation to form friendships with culturally diverse individuals when interacting with non-native speakers (Jiang et al., 2020). International students may face acculturative stress and difficulties adjusting to the host country’s environment (Smith and Khawaja, 2011), living far from their family and having to make new friends, trying new foods, and adjusting to new learning and communication styles. They must embrace local practices and traditions, often under cultural pressure or even culture shock, which is referred to as acculturation, a dynamic, complex, and multidimensional adaptation process (Berry, 2005).

In their research on the acculturation of Chinese students in the US, Lai et al. (2023) underlined that they prefer a separation acculturation strategy. International students may confront some difficulties such as social, cultural, and academic obstacles which cause them to feel acculturative stresses (Bamber, 2014). The psychological and sociological aspects of overseas students’ acculturation are often better than the expected average (Zhang and Zeng, 2023).

Researchers highlight the diversity among international students, noting that their distinct experiences, backgrounds, personal narratives, and perspectives significantly influence their multiple identities and sense of self (Eulatth Vidal and Kamp, 2025). International students face acculturative stress during their acculturation process due to the differences between the culture of their home countries and host countries (Kristiana et al., 2022), which is caused by experiencing different problems and obstacles (Berry, 2003). This research contends that although students may experience high life satisfaction, significant acculturative stress can hinder their effective adaptation to the host culture. Acculturative stress is proposed to moderate the link between life satisfaction and acculturation. Although life satisfaction typically supports better adjustment, elevated stress may drain students’ emotional and cognitive resources, limiting their ability to translate satisfaction into positive experiences (Berry, 2006). Hence, the hypothesis is set as follows:

*H4*: higher acculturative stress weakens the positive relationship between life satisfaction and acculturation.

International students usually encounter social, academic, and psychological difficulties (Lashari et al., 2023). Students from collectivistic societies, like those in Africa and Asia, may find it difficult to adjust to individualist societies, like those in North America and Europe (Sullivan and Kashubeck-West, 2015). International students who struggle with cross-cultural adaptation will have academic failure and critical mental health conditions like anxiety and despair (Anderson and Guan, 2018; Mahmood and Galloway Burke, 2018). In this regard, acculturative stress may lead to a remarkable decline in positive psychological outcomes like psychological adjustment, mental health, life satisfaction, and quality of life, as well as an increase in negative psychological outcomes like depression, psychological distress, and overall stress (Amlashi et al., 2024). Due to cultural differences and conflicts, international students may encounter inner discomfort, irritability, anxiety, and panic (Yang et al., 2021).

Research on the acculturation of international students in China highlighted some acculturative stresses such as difficulties in sociocultural adjustment and integrating with local students (Wen et al., 2018) and understanding Chinese culture and risk of cultural misunderstandings with local people, especially by African and European students (Qadeer et al., 2021). In conjunction with the findings presented by Zhu et al. (2022), this study argues that Chinese universities should address international students’ acculturation issues, promote international exchange, and develop and implement effective policies to enhance the acculturation status of international students. When international students experience high levels of acculturative stress, even those with high life satisfaction may be less inclined to recommend their institution. The positive feelings associated with life satisfaction may be overshadowed by the stress they experience in navigating the host culture.

*H5*: higher acculturative stress weakens the positive relationship between life satisfaction and institutional recommendations.

## 3 Method

### 3.1 Data collection

This study used a cross-sectional design and employed an explanatory research technique to gain a better understanding of the international students’ life satisfaction based on their experiences, and its impact on their acculturation state and institutional recommendations while considering the potential moderating influence of acculturative stress. International students experience cultural contact and learning throughout their studies in the host country. Our research defines international students in China as cultural sojourners and learners.

Data were collected using self-administered questionnaires distributed to international students with at least 1 year of study and residence in China, across 13 universities. The research purpose was explained, confidentiality was assured, and written consent was obtained. With the assistance of several moderators, printed questionnaires were distributed after classes and in dormitories between October 2024 and January 2025. Out of 297 collected questionnaires, 16 were excluded due to missing or inconsistent data, resulting in 281 valid responses for analysis.

### 3.2 Measures

This study measured international students’ academic experience with three items and accommodation experience with four items, both based on scales developed by Ammigan (2019). Health and Safety experience; and support service experience were each measured using three items designed by Chelliah et al. (2019). Sociocultural experience was assessed with four items developed by Wen et al. (2018) while discrimination experience was a three-item scale developed by Wekullo (2019).

In this study, international student life satisfaction is operationally defined as the overall contentment and positive evaluation expressed by international students regarding their academic and living conditions in China. The international students’ level of satisfaction was assessed using three items developed by Chelliah et al. (2019). This scale was designed to measure overall satisfaction among international students, had high reliability, and was employed in similar studies (Qadeer et al., 2021). Additionally, this scale supports the theoretical and grounded link between international students’ experiences and overall life satisfaction by recognizing satisfaction as a subjective, cumulative assessment of the student’s experience in the host country.

Acculturative stress was measured using 10 items extracted from Sandhu and Asrabadi (1994). This scale focuses mainly on the psychological and emotional aspects of cultural adaptation, such as language barriers, discrimination, and homesickness. This distinct focus helps avoid overlap with Stephenson (2000) acculturation scale, which measures cultural adaptation strategies rather than stressors. By clearly differentiating between acculturative stress (negative experiences) and acculturation (adaptation strategies), you minimize the risk of collinearity, ensuring that each construct contributes uniquely to your analysis of how life satisfaction impacts acculturation. This study builds on the individual-level definition of acculturation as “the experience of psychological adjustment as key life experiences that can be viewed as opportunities or impediments” (Berry, 1997).

The acculturation status of international students was assessed using the modified multigroup acculturation scale developed by Stephenson (2000). This scale has 24 items and includes two dimensions: ethnic society immersion; and dominant society immersion. Given the purpose and scope of our study, we used only the 12 items of dominant society immersion which focuses on reflecting the attitudes and behaviors related to four domains: language, interaction, media, and food. Acculturation involves adapting to the host culture, making it logical to focus on dominant society immersion—language use, social interactions, media habits, and food preferences. Aligned with Berry (1997) model, the 12-item subscale better reflects acculturation behaviors than the ethnic society immersion subscale, which emphasizes heritage culture.

Our research defines institutional recommendation as the international student’s willingness to promote their university to prospective international students. Institutional recommendation was measured using three items adapted from Mavondo et al. (2004) and Chelliah et al. (2019). The measurement items for each construct, presented in Appendix 1, were assessed using a 5-point Likert scale.

### 3.3 Sample characteristics

Table 1 shows the study sample characteristics. The valid sample included 281 students (46%) from Asia, (40%) from Africa, (7%) from Europe, (4%0 from Oceania) and 3% from the Americas. China has 253,177 international students, mainly from other Asian countries (59%) and African countries (18.5%) (Ministry of Education, 2023). According to the self-reported data, 68% of the respondents are males, with 42% aged between 21–25 years and 27% between 26–30 years. The majority of international students are pursuing undergraduate degrees (72%), primarily in the fields of Social Sciences and Humanities (54%) and Science, Technology, Engineering, and Mathematics (37%).

**Table 1:** International students’ sample characteristics (281).

Variable	Category	Frequency	Percent
Age	≤20	48	17
	21–25	118	42
	26–30	76	27
	≥31	39	14
Gender	Male	192	68
	Female	89	32
Continent	Asia	129	46
	Africa	113	40
	Europe	19	7
	Oceanian	12	4
	Americas	8	3
Educational level	Undergraduate	202	72
	Postgraduate and above	79	28
Major	Science, Technology, Engineering, and Mathematics	104	37
	Social Sciences and Humanities	152	54
	Professional and Applied Sciences	25	9

## 4 Results

### 4.1 Correlation analysis

The processed dataset met preliminary regression assumptions, enabling robust analysis. Normality was confirmed using the Shapiro–Wilk test (appropriate for samples under 2000), which was non-significant (*p* > 0.05), indicating a normal distribution. The zero-order correlations among study variables shown in Table 2 revealed a positive correlation between international student positive experiences and life satisfaction: sociocultural experience (*r* = 0.30, *p* < 0.01), accommodation experience (*r* = 0.45, *p* < 0.01), health and safety (*r* = 0.42, *p* < 0.01), social service support (*r* = 0.35, *p* < 0.01). On the other hand, both discrimination experience (*r* = −0.24, *p* < 0.05) and academic experiences (*r* = −0.27, *p* < 0.05) had a negative correlation with international students’ life satisfaction. Additionally, international student life satisfaction is positively correlated with their acculturation (*r* = 0.41, *p* < 0.01) and institutional recommendation (*r* = 0.44, *p* < 0.01), indicating that students with higher life satisfaction are more likely to acculturate and recommend their institutions. The correlation findings showed that the study constructs are linear and significantly related. Additionally, the VIF and tolerance values were used to test the potential multicollinearity. The reported VIF value is 1.283 which is higher than the 0.5 threshold, and the tolerance value of 0.782 which is higher than the 0.2 threshold, ensuring there are no multicollinearity concerns (Hair et al., 2010).

**Table 2:** Zero-order correlations among study variables.

Construct	Mean	SD	1	2	3	4	5	6	7	8	9	10
1. ACC	3.74	0.513	1									
2. INS REC	3.81	0.562	0.22*	1								
3. LF SAT	4.07	0.591	0.41**	0.44**	1							
4. ACC STR	3.22	0.437	−0.28*	−0.38*	−0.42*	1						
5. ACDEXP	3.19	0.423	−0.21*	−0.23*	−0.27*	0.31*	1					
6. SC EXP	3.51	0.462	0.33**	0.31*	0.30**	−0.26*	0.30**	1				
7. ACC EXP	4.23	0.645	0.46**	0.49**	0.45**	−0.47*	0.43*	0.34**	1			
8. H&S EXP	4.27	0.673	0.49**	0.44**	0.42**	−0.25**	0.40*	0.28*	0.41**	1		
9. SS EXP	4.11	0.612	0.39**	0.41*	0.35**	−0.43**	0.46**	0.37*	0.46**	0.54**	1	
10. DIS EXP	3.08	0.416	−0.28*	−0.39**	−0.24*	0.35*	−0.28*	−0.42**	−0.29**	−0.24*	−0.31**	1

ACD EXP, academic experience; SC EXP, socio-cultural experience; ACC EXP, accommodation experience; H&S EXP, health and safety experience, SS EXP, support service experience; DIS EXP, discrimination experience; LF SAT, life satisfaction; ACCUL, acculturation; INST REC, institutional recommendation; ACC STR, acculturative stress. *p < 0.05, **p < 0.01.

### 4.2 Regression results

Hierarchical regression was conducted using SPSS 26, incorporating both direct and moderation analyses. Prior to testing moderation, the conditions outlined by Preacher et al. (2006) were followed to ensure proper analytical procedures. First, variables are mean-centered to minimize multicollinearity. Second, it is essential to confirm that the moderator is significantly correlated with the dependent variable. Third, the interaction term was assessed by multiplying the values of the moderator and the independent variable, ensuring that the resulting values are greater than zero. Finally, a hierarchical regression analysis with interaction terms was undertaken to test the moderating effect. A moderating effect is confirmed when the variance explained (*R*^2^) by the moderated model is both substantial and greater than the variance explained (*R*^2^) in the unmoderated model (Preacher et al., 2006).

Before testing hypotheses H1a-H1f, multiple hierarchical regression was performed to report the specific impact of the control variables and the effect of the six international students’ experiences on their life satisfaction. Table 3 shows the multiple hierarchical regression findings. It is evident that Model 1, including control variables only, explained only 0.10.8% of the variance in life satisfaction.

**Table 3:** Hierarchal multiple regression.

Construct	Model 1				Model 2			
	*Β*	*T*	*p*-value	95% CI	*β*	*T*	*p*-value	95% CI
Age	0.102	2.76	0.025	(0.03, 0.17)	0.089	2.41	0.037	(0.01, 0.16)
Gender	0.054	1.46	0.145	(−0.04, 0.12)	0.059	1.61	0.109	(−0.01, 0.13)
Continent	0.048	1.31	0.191	(−0.02, 0.13)	0.042	1.14	0.254	(−0.03, 0.11)
Education Level	0.034	0.92	0.358	(−0.03, 0.10)	0.021	0.57	0.570	(−0.05, 0.09)
Major	−0.025	−0.67	0.605	(−0.09, 0.04)	−0.011	−0.29	0.772	(−0.08, 0.06)
ACD EXP					−0.113	−2.83	0.005	(−0.19, −0.03)
SC EXP					0.254	6.85	<0.001	(0.18, 0.32)
ACC EXP					0.238	6.42	<0.001	(0.16, 0.30)
H&S EXP					0.285	7.71	<0.001	(0.21, 0.35)
SS EXP					0.192	5.18	<0.001	(0.11, 0.26)
DIS EXP					−0.038	−1.04	0.300	(−0.10, 0.03)
*R* ^2^	0.018				0.471			
*R*^2^ change	0.018				0.453			
*F*	3.107		0.009		35.947		<0.001	
Durbin Watson					1.693			

ACD EXP, academic experience; SC EXP, socio-cultural experience; ACC EXP, accommodation experience; H&S EXP, health and safety experience; SS EXP, support service experience; DIS EXP, discrimination experience; LF SAT, life satisfaction; ACCUL, acculturation; INST REC, institutional recommendation; ACC STR, acculturative stress.

DV, life satisfaction.

With the inclusion of the six international student experiences, Model 2 showed that the foreign students’ life satisfaction was negatively associated with their academic experience (*β* = −0.113, t = −2.83, *p* = 0.005), positively linked with their health and safety experience (*β* = 0.285, *t* = 5.92, <0.001), accommodation experience (*β* = 0.238, *t* = 4.88, <0.001), sociocultural experience (*β* = 0.254, *t* = 5.31, <0.001), and support service experience (*β* = 0.192, *t* = 4.05, <0.001).

On the other hand, discrimination experience had no statistically significant effect on the life satisfaction of international students (*β* = −0.038, *t* = −0.83). Among the control variables, only international students’ age was positively related to the life satisfaction of international students (*β* = 0.102, *t* = 2.76, *p* = 0.025). The overall model demonstrated a substantial improvement in explanatory power with the inclusion of independent variables, increasing from 10.8% (*R*^2^ = 0.018, *F* = 3.107) in Step 1 to 47.1% (*R*^2^ = 0.471, *F* = 35.947, *p* = <0.001) in Step 2, highlighting the importance of these experiential factors in shaping life satisfaction.

The hierarchal regression findings shown in Table 4 revealed that international students’ life satisfaction positively influences their acculturation state (*β* = 0.461, *t* = 11.85, *p* < 0.001) and their institutional recommendation (*β* = 0.382, *t* = 9.25, <0.001). These findings indicated that higher levels of life satisfaction are strongly associated with better acculturation and positive institutional recommendation outcomes among international students in China. Such results underscore the importance of understanding the antecedents and consequences of international students’ life satisfaction. The moderating regression revealed that acculturative stress weakens the positive connection between life satisfaction and acculturation (*β* = −0.122, *t* = 4.19, <0.001).

**Table 4:** Moderated regression analysis.

Moderation effect	DV: acculturation						DV: institutional recommendation					
	COEFF	SE	*t*	*p*-value	LLCI	ULCI	COEFF	SE	*t*	*p*-value	LLCI	ULCI
Age	0.078	0.063	1.82	0.07	0.03	0.15	0.064	0.052	1.46	0.15	−0.02	0.15
Gender	0.058	0.052	1.35	0.18	−0.02	0.14	0.052	0.043	1.21	0.23	−0.03	0.13
Continent	0.032	0.044	0.73	0.47	−0.05	0.11	0.045	0.041	1.05	0.30	−0.03	0.12
Education Level	0.019	0.044	0.43	0.67	−0.06	0.10	0.021	0.043	0.49	0.62	−0.06	0.10
Major	−0.015	0.044	−0.34	0.73	−0.10	0.07	−0.009	0.043	−0.21	0.83	−0.09	0.07
LF SAT	0.461	0.041	11.85	<0.001	0.37	0.51	0.382	0.045	9.25	<0.001	0.29	0.46
ACC STR	−0.172	0.038	4.19	<0.001	−0.09	0.23	−0.112	0.035	2.35	0.02	−0.09	0.23
Int_1(LF SAT*ACC STR)	−0.122	0.035	−3.487	<0.001	−0.17	−0.05	−0.038	0.043	−0.89	0.37	−0.12	0.04
*R* ^2^	0.286						0.137					
*F*	28.22			<0.001			13.15			<0.001		

This finding indicates that the positive impact of life satisfaction on acculturation diminishes when individuals experience higher levels of acculturative stress. Regarding model fit, the predictors explained approximately 28.6% of the variance in acculturation, with an overall model that was statistically significant (*F* = 28.22, at Sig 0.000). The interaction between life satisfaction and acculturative stress was negative and statistically significant (*β* = −0.122, *p* < 0.05), indicating that acculturative stress weakens the positive effect of life satisfaction on acculturation. However, the magnitude of the moderating effect was relatively small.

**Figure 2:**
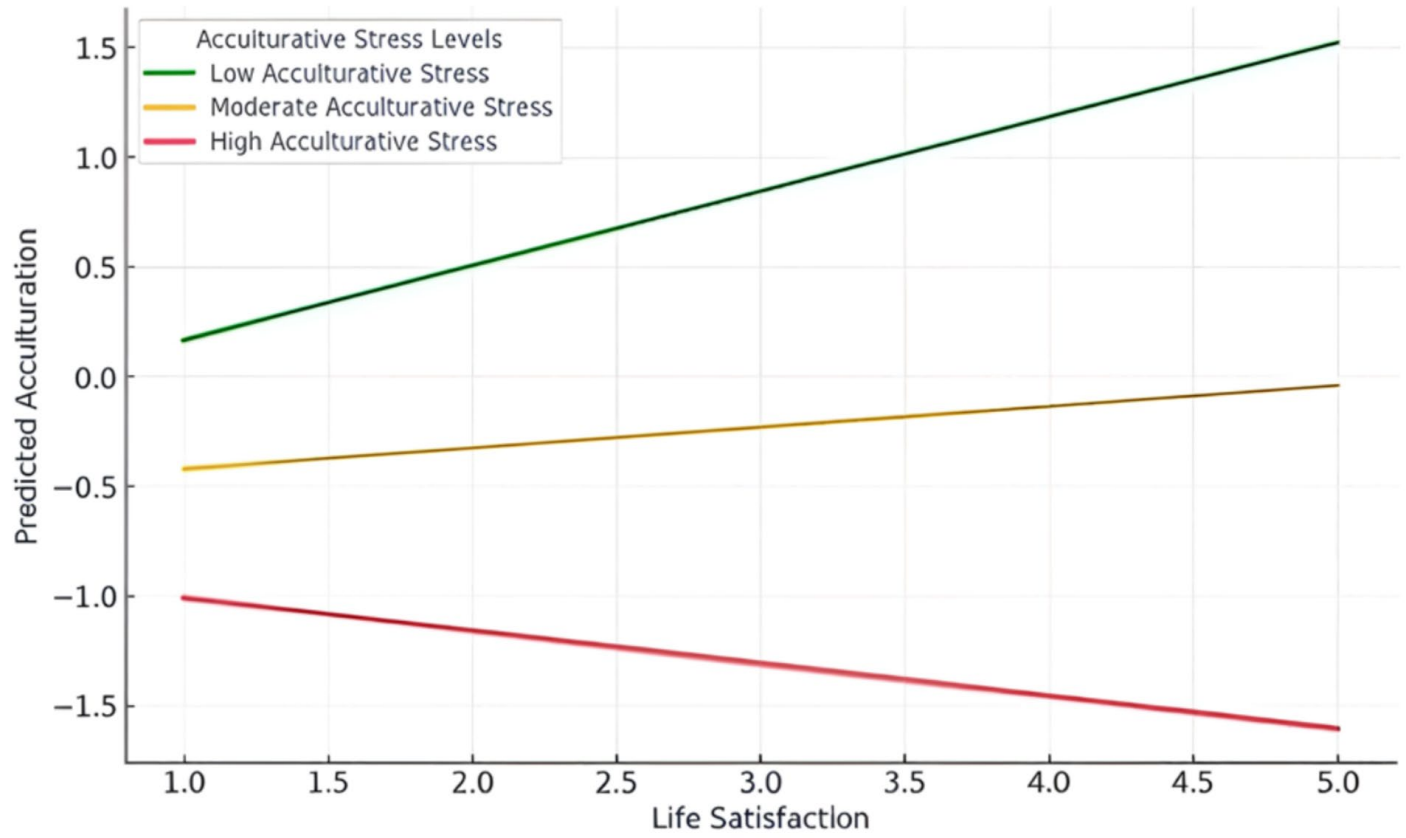
The moderating role of acculturative stress.

Finally, acculturative stress had no significant moderating impact on the relationship between life satisfaction and institutional recommendation (*β* = −0.038, *t* = −0.89). This indicates that acculturative stress does not moderate the relationship between life satisfaction and institutional recommendation (Figure 2).

## 5 Discussion

Sub-hypotheses H1a to H1f examined the impact of six key experiences—academic, socio-cultural, accommodation, health and safety, support services, and discrimination—on international students’ life satisfaction. Regression results revealed that academic experience had a negative effect on life satisfaction (H1a), supporting the view that many Chinese universities face challenges in providing balanced development for international students in areas such as tutor allocation, structured Chinese language instruction, academic research quality, research platforms, and data accessibility (Beregovaya et al., 2020). Supporting academic adaptation is essential for students’ success and well-being (Al Juboori et al., 2025). Due to the diverse backgrounds and cultures of international students, Chinese universities face challenges in standardized management, including inconsistent student quality, lenient training processes, low assessment standards, and broad teaching practices. These issues may undermine the global reputation and status of Chinese higher education (Poole et al., 2022).

In contrast, regression results showed that socio-cultural, accommodation, health and safety, and support service experiences (H1b–H1e) significantly and positively influenced life satisfaction, while discrimination experiences (H1f) had no significant effect. Studies on international students’ life satisfaction have shown that their satisfaction increases when their educational experience exceeds expectations (Appleton-Knapp and Krentler, 2006), particularly when they encounter positive aspects of learning, housing, and support services (Ammigan and Jones, 2018). Besides social and cultural issues, international students encounter academic challenges in their new educational contexts (Choy et al., 2021; Lai et al., 2023).

Despite some studies identified some concerns related to evaluating the quality of accommodation such as lack of single rooms, slow internet, outdated dorm facilities, and improper behavior from dorm staff (Tian and Lowe, 2018; Wen and Hu, 2019), Our findings showed that international students are satisfied with their accommodation experiences (Ammigan and Jones, 2018). In conjunction with extant literature (Arambewela and Hall, 2009; Chelliah et al., 2019; Qadeer et al., 2021), our study found that international students’ health and safety experiences positively affect their life satisfaction in China.

Research indicates a moderate-to-strong link between acculturative stress and depressive symptoms among international university students (Çimşir and Ünlü Kaynakçı, 2024). Furthermore, after an initial period of excitement about exploring a new culture, the novelty may fade, and international students must manage daily challenges in an unfamiliar environment (Smith and Khawaja, 2011; Xiong et al., 2025). Acculturative stress was negatively correlated with academic adaptation (Al Juboori et al., 2025).

Contrary to Wen et al. (2018), our findings indicate that international students’ sociocultural experiences positively influence their life satisfaction. Engaging with the host culture through social activities, friendships, and cultural events enhances emotional well-being and reduces isolation, ultimately fostering greater life satisfaction.

The findings showed that support services from international student offices positively influenced students’ life satisfaction. However, these results diverge from those reported in prior research (Ding, 2016; Tian and Lowe, 2018; Wen and Hu, 2019). Our findings can be justified based on the fact that previous studies might have examined support services that were inadequate, poorly implemented, or mismatched to students’ needs, leading to frustration and reduced life satisfaction.

Despite the possibility that international students in China may face discrimination (Tian and Lowe, 2018), our findings showed that it has no significant influence on their life satisfaction. Such findings suggest that international students developed resilience and effective coping mechanisms that reduced discrimination’s negative impact. Strong social support systems or prioritizing factors like academic success might also buffer its effects. Additionally, the level of discrimination experienced could have been low or infrequent, or students might have normalized it based on cultural expectations.

For hypotheses H2 and H3, the findings showed that international students’ life satisfaction had a positive influence on both acculturation state and institutional recommendation. In conjunction with recent studies on the acculturation of international students (Jiang et al., 2020; Peng et al., 2023), our research underlined that it is positively related to life satisfaction. These findings may indicate that international students who experience high life satisfaction are more likely to feel emotionally stable, motivated, and positive about their overall experience, which facilitates smoother acculturation. Satisfied students are better equipped to engage with the host culture, build social connections, and adapt to new norms, enhancing their acculturation state. Similarly, our findings showed that international students’ life satisfaction had a positive influence on their favorable institutional recommendations (Mavondo et al., 2004; Ammigan, 2019; Chelliah et al., 2019). These results suggest that high life satisfaction reflects a positive institutional experience, increasing the likelihood of recommending the institution. Satisfied students link their well-being to the support and environment provided, making life satisfaction a key driver of successful adaptation and positive recommendations.

Testing the moderating role of acculturative stress (H4) revealed that it weakens the positive relationship between life satisfaction and international students’ acculturation. This finding aligns with existing literature on the negative effects of acculturative stress (Smith and Khawaja, 2011; Driscoll and Torres, 2022; Zhang and Zeng, 2023). Our results further suggest that even students with high life satisfaction may struggle to adapt when stress levels are elevated. This highlights the need for universities to complement well-being initiatives (e.g., counseling services) with structural reforms, such as streamlining administrative procedures, to address the root causes of acculturative stress.

In contradiction with similar studies on the negative consequences of acculturative stress (Zhu et al., 2022; Amlashi et al., 2024), our study revealed that it has no moderating effect on the relationship between life satisfaction and institutional recommendations (H5). Such findings may indicate that even if students experience acculturative stress, their satisfaction with the institution’s services and support might remain high, leading to positive recommendations. Second, students may separate their struggles with acculturation from their evaluation of the institution, focusing instead on its tangible benefits. Finally, the sample might have included resilient students who, despite experiencing acculturative stress, maintained a positive view of the institution due to its role in providing stability or assistance during their adaptation process. The results of our hypotheses testing provide remarkable implications to theory and practice as explained in subsequent sections.

## 6 Implications

The empirical findings from this study hold significant theoretical and practical implications, contributing to academic knowledge while offering actionable insights for enhancing international students’ satisfaction, acculturation and institutional recommendations.

### 6.1 Theoretical implications

This study approved the relevance and applicability of the SET by highlighting the reciprocal nature of relationships and exchanges between international students and their host institutions. SET posits that individuals engage in relationships based on perceived rewards and costs, and your findings align with this by demonstrating that positive experiences (rewards) enhance satisfaction, which in turn fosters acculturation and positive institutional recommendations. International students participate in academic, economic, social, and cultural endeavors, fostering interactions with host communities within university environments and in the broader society (Ranabahu and De Silva, 2024). However, acculturative stress imposes a cost that weakens the link between satisfaction and acculturation, highlighting the delicate balance between rewards and challenges in the acculturation process. The study extends the SET by emphasizing the role of contextual stressors in shaping the exchange dynamics within cross-cultural educational settings.

Building on Berry’s acculturation theory, this study emphasized the balance between cultural maintenance, participation in the host society, and the stressors encountered during acculturation. Our findings showed that positive experiences boost satisfaction, which in turn facilitates acculturation and institutional recommendations—indicators of successful integration. However, the moderating role of acculturative stress underscores the challenges students face in adjusting while maintaining well-being, consistent with Berry’s view of stress as a barrier to adaptation.

### 6.2 Practical implications

Our empirical findings suggest that improving international students’ life satisfaction requires institutional policies focused on socio-cultural integration through cultural events and inclusive campus environments. Affordable, safe, and comfortable accommodation is essential, along with enhanced health and safety measures such as accessible healthcare and robust campus security. Support services—academic advising, mental health counseling, and language assistance—should be expanded and actively promoted. Furthermore, the negative impact of academic experiences on life satisfaction highlights the need for targeted interventions to reduce academic stress, including tailored support like tutoring, language assistance, and mental health resources.

University leaders, department heads, government agencies, and non-profits increasingly rely on international student behavior and mobility to shape policies and strategic plans for diverse global student populations (Luo et al., 2023). A recent systematic review highlights the need for future research to establish strong theoretical foundations and explore emerging trends, processes, and challenges in international student mobility within today’s dynamic global context (Dos Santos et al., 2024). Policies should also encourage inclusive, flexible teaching methods to accommodate diverse learning styles and cultural backgrounds.

To strengthen the positive impact of life satisfaction on acculturation and institutional recommendation, institutions should prioritize policies that support international students’ well-being. This includes fostering a welcoming environment through cultural integration programs, peer mentorship, and cross-cultural social events. Actively seeking student feedback can help identify areas for improvement, while inclusivity policies such as diversity training for staff and students can create a more supportive campus atmosphere.

Finally, institutions should adopt policies that reduce stress and support international students’ acculturation, such as cultural orientation workshops, language classes, and stress management seminars. Social support programs can help students cope with acculturative stress and improve their adjustment (Zheng and Ishii, 2023). The acculturation of international students is influenced by their engagement with formal academic systems and informal social networks (Ranabahu and De Silva, 2024). Fostering an inclusive campus through diversity training and cultural awareness initiatives can enhance students’ adaptability and sustain high life satisfaction.

## 7 Limitations and further research

Our findings may have limited applicability due to certain constraints. First, the sample of 281 international students, mainly from Asian and African countries, may not represent the broader international student population in China. For example, East Asian students often report higher satisfaction than those from more culturally distant backgrounds (Alemu and Cordier, 2017). Hence, future research may focus on a specific race or use comparative studies.

Second, relying on self-reported surveys may introduce bias and affect the findings. Accordingly, future research may prioritize using longitudinal studies to explore temporal relationships, conducting evidence-based interventions to apply findings in practice, and using mixed-methods approaches to gain deeper insights into the dynamics of life satisfaction, acculturation, and institutional recommendation over time.

Third, institutional variations such as university policies and support services were not systematically analyzed, despite their potential impact. Future research should explore how improvements in socio-cultural, accommodation, health and safety, and support service experiences can enhance international students’ life satisfaction. Additionally, it should investigate why academic experiences negatively affect satisfaction and identify solutions, such as curriculum changes, academic support, or faculty training.

Finally, although acculturative stress was identified as a moderating factor, its multidimensional nature was not thoroughly examined, which may have led to an oversimplification of its impact. Future research should explore targeted interventions aimed at reducing specific dimensions of acculturative stress and further investigate its nuanced moderating role in the relationship between life satisfaction and acculturation.

## Data Availability

The original contributions presented in the study are included in the article/Supplementary material, further inquiries can be directed to the corresponding author.
