# Supplementary material for: The impact of life satisfaction on acculturation and institutional recommendation among international students in China: does acculturative stress matter?
Source: Front Psychol. 2025 Jul 2;16:1584210. doi: 10.3389/fpsyg.2025.1584210 (PMC12269862; doi:10.3389/fpsyg.2025.1584210)
Supplement: Supplementary file 2 [file Table_2.DOC]

The Impact of Life Satisfaction on Acculturation and Institutional Recommendation Among Foreign Students in China. The Moderating Role of Acculturative Stress.

**Measures Scales**

| **Accommodation Experience** (Santos, 2018; Ammigan, 2019) | 1 | 2 | 3 | 4 | 5 |
| --- | --- | --- | --- | --- | --- |
| All of the necessary facilities have been provided at my accommodation |  |  |  |  |  |
| The location of my accommodation is convenient for going to school |  |  |  |  |  |
| A good environment is provided at my accommodation |  |  |  |  |  |
| Access to suitable accommodations has been given |  |  |  |  |  |
| **Academic experience** (Ammigan, 2019). |  |  |  |  |  |
| The academic staff have a good command of English |  |  |  |  |  |
| Lecturers are experts |  |  |  |  |  |
| I have easy access to the academic staff |  |  |  |  |  |
| **Health and Safety** (Chelliah et al. 2019) |  |  |  |  |  |
| A healthy and safe environment is provided by the university |  |  |  |  |  |
| Student health issues are responded to in a timely fashion |  |  |  |  |  |
| In general, there is a safe environment for international students inside or outside of campus |  |  |  |  |  |
| **Support services experience** (Chelliah et al. 2019) |  |  |  |  |  |
| The staffs from the international student office listen to the students’ concerns |  |  |  |  |  |
| The staffs from the international student office perform their duties in friendly manner |  |  |  |  |  |
| The staffs from the international student office are always willing to help a student to ease his or her discomfort |  |  |  |  |  |
| **Sociocultural experience (Wen et al., 2018)** |  |  |  |  |  |
| I have become accustomed to the pace of life in China |  |  |  |  |  |
| I understand the Chinese value system |  |  |  |  |  |
| I have adapted to the local etiquette |  |  |  |  |  |
| It is easy for me to make local friends |  |  |  |  |  |
| **Discrimination experience (Wekullo, 2019; Harrison, 2010)** |  |  |  |  |  |
| I never face a discriminatory attitude from faculty |  |  |  |  |  |
| I never face a discriminatory attitude from classmates or other students |  |  |  |  |  |
| I never face a discriminatory attitude from local people |  |  |  |  |  |
| **Student satisfaction (Chelliah et al. 2019)** |  |  |  |  |  |
| Based on all of the experiences, I feel that attending this university was the right decision |  |  |  |  |  |
| I am happy with the learning support given by the lecturers |  |  |  |  |  |
| In general, I am satisfied with the facilities |  |  |  |  |  |
| **Acculturative Stress (**Mena, Padilla, and Maldonado in 1987; Sandhu & Asrabadi, 1994**)** |  |  |  |  |  |
| "I feel stressed because I have difficulty communicating in Chinese." |  |  |  |  |  |
| "I feel stressed because I have experienced discrimination or unfair treatment as a foreign student in China." |  |  |  |  |  |
| "I feel stressed because I miss my home country, family, or friends while studying in China." |  |  |  |  |  |
| "I feel stressed because I am unsure how to balance my own cultural values with Chinese cultural norms." |  |  |  |  |  |
| "I feel stressed because I find it hard to make friends or feel accepted by Chinese students or locals." |  |  |  |  |  |
| "I feel stressed because I struggle to meet academic expectations or adapt to the teaching style in Chinese universities." |  |  |  |  |  |
| "I feel stressed because I worry about being judged or rejected by others due to my foreign background." |  |  |  |  |  |
| "I feel stressed because I find it difficult to adapt to Chinese customs, traditions, or daily life." |  |  |  |  |  |
| "I feel stressed because my family and I have disagreements about my decision to study in China." |  |  |  |  |  |
| "I feel stressed because I am uncertain about my future career or life in China after graduation." |  |  |  |  |  |
| **Acculturation Status** Stephenson (2000) |  |  |  |  |  |
| I am informed about current affairs in China |  |  |  |  |  |
| I feel totally confident with Chinese people |  |  |  |  |  |
| I have many Chinese acquaintances |  |  |  |  |  |
| I feel home in China |  |  |  |  |  |
| I feel accepted by Chinese |  |  |  |  |  |
| I know how to prepare Chinese foods |  |  |  |  |  |
| I regularly read a Chinese newspaper |  |  |  |  |  |
| I speak Chinese at home |  |  |  |  |  |
| I am familiar with important people in Chinese history |  |  |  |  |  |
| I think in Chinese |  |  |  |  |  |
| I speak Chinese with my classmates, supervisor or instructor. |  |  |  |  |  |
| I like to eat Chinese foods |  |  |  |  |  |
| **Institutional Recommendation** (Chelliah et al. 2019; Mavondo, et al., 2004) |  |  |  |  |  |
| I will recommend my university to other international students for their future studies |  |  |  |  |  |
| This university will be the first choice when I need to recommend an institution to others |  |  |  |  |  |
| Based on my satisfaction with the university, I am more likely to recommend it to future students |  |  |  |  |  |
